# Depressive and anxiety symptoms, their predictors, and pregnancy outcomes among Omani pregnant women: A prospective cohort study

**DOI:** 10.1177/17455057261476574

**Published:** 2026-08-10

**Authors:** Mohammed Al Sinani, Samir Al-Adawi, Mohammed Al Meqbaali

**Affiliations:** 1 Department of Nutrition, Al-Buraimi Hospital, Ministry of Health, Al Buraimi, Oman; 2 Department of Metabolism, Digestion and Reproduction, Faculty of Medicine, 216729 Imperial College London, London, UK; 3 Department of Behavioral Medicine, College of Medicine and Health Sciences, 108707 Sultan Qaboos University, Muscat, Oman; 4 455210 Nursing, Fatima College of Health Sciences, Al Ain, UAE

**Keywords:** maternal mental health, EPDS, Omani women, pregnancy, preterm birth, low birth weight, screening

## Abstract

**Background:**

Maternal depression during pregnancy is a major global health concern that affects both mothers and infants. However, limited studies have examined maternal depression across pregnancy stages in Arabic-speaking populations, where fertility rates are high.

**Objectives:**

This study aims to evaluate the relationship between antenatal depression and anxiety symptoms during the early (8-12 weeks) and later stages (24-28 weeks) of pregnancy and their effects on maternal and neonatal outcomes among Omani women.

**Design:**

Prospective cohort study.

**Methods:**

A prospective cohort design was used, involving 302 pregnant Omani women receiving antenatal care at Al Buraimi Hospital. Eligible participants were aged 18–45 years, between 8–12 weeks of gestation and expected to continue care at the same clinic. Depression and anxiety were measured at both stages using the Arabic version of the Edinburgh Postnatal Depression Scale (EPDS) and the EPDS-3A subscale. Statistical analyses, including chi-square tests and logistic regression, were used to examine associations between anxiety, depression, and pregnancy outcomes. Multiple regression analyses controlled for maternal age, marital status, parity, pre-pregnancy BMI, and household income.

**Results:**

High levels of depressive and anxiety symptoms were identified, particularly as pregnancy advanced. Among 302 pregnant Omani women, the prevalence of depressive symptoms was 29.8% and anxiety symptoms was 24.8% in early pregnancy. Women with elevated EPDS scores had higher risks of caesarean delivery, low birth weight, and preterm birth. Elevated anxiety was associated with greater maternal distress and poorer neonatal outcomes.

**Conclusion:**

Findings highlight the importance of integrating mental health screening into routine antenatal care in Oman. Early identification and management of depression and anxiety may reduce adverse outcomes for mothers and infants. Further research should investigate barriers to mental health services for pregnant women and the long-term developmental effects of antenatal depression on children.

## 1. Introduction

Emotional disturbances during pregnancy, including antenatal depression and anxiety, are increasingly recognized as significant public health concerns due to their impact on maternal well-being and child development.^1^ Antenatal depression is among the leading causes of disability in pregnant women globally, especially in low-income settings.^2^ Although prevalence rates vary between countries, antenatal depression is generally more common than postpartum depression.^3^

Globally, approximately 15.6% of women experience prenatal depression.^4^ Depressive symptoms can occur throughout pregnancy, although published studies have reported variable prevalence across trimesters without a clearly consistent temporal pattern.^5^ Meta-analytical data show that depression affects 7.4% of women in the first trimester, 12.8% in the second, and 12% in the third.^6^ Clinical depression is diagnosed in 10–20% of pregnant women, while over a quarter report depressive symptoms.^7^

In the Arab world, the prevalence of maternal depression ranges between 10% and 37%.^8^ Among Gulf countries, the highest rate is reported in Bahrain (37%)^9^; followed by the UAE (10–20%),^10,11^ Kuwait (11.7%)^12^; and Qatar (17.6%).^13^ In Oman, antenatal depression is increasingly observed in both clinical practice and community-based research.^14^ One national study found that 12% of pregnant women were at risk of postnatal depression, while another reported a 24.3% prevalence of antenatal depression among 959 pregnant women higher than rates in several neighboring countries.^15^

Risk factors for antenatal depression are multifactorial and include low education, poor socioeconomic status, limited social support, marital conflict, intimate partner violence, and history of mental health issues.^16^ Biological, hormonal, and medical factors may further increase vulnerability to depressive symptoms during pregnancy.^17,18^ In Oman, a recent study identified unplanned pregnancy and marital discord as key predictors of depression during pregnancy.^15^

Antenatal depression is strongly associated with adverse outcomes such as preeclampsia (PE), caesarean section (CS), suicidal ideation, and increased risk of postpartum depression.^19^ It also contributes to preterm delivery, low birth weight, neonatal intensive care admissions, stillbirth, and delayed child development.^20^ This psychiatric condition is considered to have a multidimensional phenotype influenced by psychosocial and biological determinants.^21^ Notably, a prior history of depression or conditions like premenstrual dysphoric disorder, along with inadequate emotional support, significantly increases the risk.^22^

Despite its impact, antenatal depression often goes undiagnosed and untreated, leading to worsened health outcomes.^23^ Enhancing awareness, implementing routine screening, and providing timely interventions can significantly improve maternal and child health.^24,25^

In addition to depression, anxiety symptoms during pregnancy are increasingly recognised as a significant public health concern, with potential adverse effects on both maternal well-being and pregnancy outcomes. Evidence suggests that antenatal anxiety is associated with outcomes such as preterm birth and low birth weight. Despite this, anxiety symptoms are less frequently assessed in maternal health research, particularly in Middle Eastern populations.^20^

**Figure 1.**
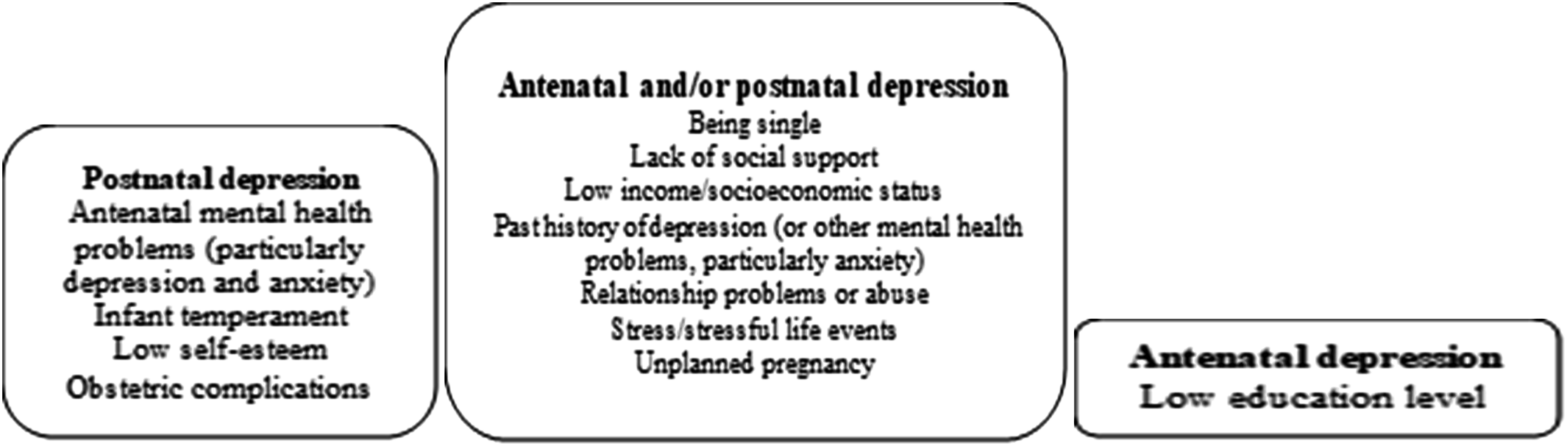
The antenatal depression and influencing factors.

This study aims to assess the prevalence of antenatal depression and anxiety among pregnant Omani women, identify associated risk factors, and explore how maternal behaviors and characteristics at two gestational time points (8–12 and 24–28 weeks) are associated with these symptoms. It also investigates the impact of these mental health conditions on maternal and neonatal outcomes to inform future preventive strategies in Oman (Figure 1).

## 2. Methods

This prospective cohort study was conducted at Al Buraimi Hospital in northern Oman, targeting pregnant Omani women residing in Al Buraimi Governorate. A total of 302 pregnant Omani women were recruited and included in the final baseline analysis. A total of 300 participants were recruited from antenatal clinics affiliated with both primary and secondary healthcare services in the region. This prospective cohort study recruited pregnant Omani women at 8–12 weeks of gestation. The same participants were subsequently followed and reassessed at 24–28 weeks of gestation for depressive and anxiety symptoms and related outcomes. The study aimed to assess antenatal depression and anxiety and their associations with maternal and neonatal outcomes. Data collection was conducted between February 2019 and February 2021.

Participants were eligible if they were Omani nationals, aged 18–45, with a singleton pregnancy between 8 and 12 weeks of gestation and committed to continuing antenatal care at Al Buraimi Hospital. Women with severe health conditions (e.g., cancer, infectious diseases, psychotic or neurological disorders) or those who were non-Omani were excluded to reduce potential confounding factors. Only women aged 18 years and above were eligible; therefore, no minors were included in the study Written informed consent was obtained from all participants prior to enrolment in the study.

Data were collected from electronic medical records, covering antenatal, intrapartum, and postpartum periods. Additional information was gathered through structured online questionnaires using Qualtrics software. These questionnaires captured socio-demographic and clinical characteristics, such as maternal age, parity, marital status, education, income, employment status, physical activity, and history of mental health disorders (see supplementary materials).

A priori power analysis was conducted to determine the required sample size based on G*Power software, targeting a medium effect size (ƒ=0.3), α=0.05, and power=0.95.^26^ Based on an annual pregnancy rate of 1,800 at the hospital,^27^ 138 participants were required. To account for potential non-response and missing data (estimated at 40%), the sample size was increased to 300 to enhance reliability and generalizability.

To assess postnatal depression and anxiety, the Arabic version of the Edinburgh Postnatal Depression Scale (EPDS), validated in Oman and other Arab countries,^9,11,13^ was administered at 8–12 and 24–28 weeks of pregnancy (see supplementary materials). Depression was classified as low risk (EPDS < 10), possible (10–12), or probable (≥13). The EPDS 3-item anxiety subscale (3A-EPDS) was used to evaluate anxiety, with a cut-off score of ≥6 indicating significant symptoms, which provides an indication of anxiety symptoms but does not replace a dedicated validated anxiety assessment tool.

Depressive symptoms were assessed using the Arabic version of the Edinburgh Postnatal Depression Scale (EPDS), which has been previously validated in Arabic-speaking populations and used in Oman. The sociodemographic questionnaire was reviewed by experts and pilot-tested prior to data collection.

Statistical analysis was conducted using SPSS Version 21. Descriptive statistics summarized the study population, and inferential analyzes included Pearson’s chi-square tests and logistic regression to assess associations between depressive and anxiety symptoms and pregnancy outcomes. Variables with p ≤ 0.05 in univariate analyzes were entered into multiple logistic regression models using the entry and removal method to control potential confounders. These included maternal demographics, health behaviors, and pre-pregnancy BMI. Variables were entered simultaneously using the entering method, followed by sequential manual removal of non-significant variables based on Wald statistics and model contribution.

Analyses were conducted using a complete-case approach. Participants with missing data for relevant variables were excluded from the respective analyses. Missing data were not imputed. Ethical approval was granted by the Oman Ministry of Health’s Ethics and Research Committee (MoH/CSR/19/9668), and the study adhered to the principles of the Declaration of Helsinki (see supplementary materials). This study was reported in accordance with the STROBE Statement.

## 3. Results

### 3.1. Prevalence and predictors of antenatal depressive symptoms

A total of 302 women were recruited at baseline. Of these, 272 completed follow-ups at 24–28 weeks, and 242 had complete data for selected analyses. Prenatal depression, measured using the Edinburgh Postnatal Depression Scale (EPDS), was prevalent among the study population, with mean scores of 10.34 and a range of 0 to 25. Based on an EPDS cutoff score of ≥13, the prevalence of antenatal depression was 29.8% at 8–12 weeks and 30.5% at 24–28 weeks of gestation.

Bivariate analysis (Table 1) revealed significant associations between certain sociodemographic and clinical factors and antenatal depression at different pregnancy stages. At 8–12 weeks, housing tenure was significantly associated with depression (χ^2^ (2) = 7.72, p = 0.021), showing a small-to-medium effect (Cramér’s V = 0.18). A strong association was also observed between a history of depression and current symptoms at this stage (χ^2^ (1) = 21.96, p < 0.001), with a large effect size (Cramér’s V = 0.27).

**Table 1.** Associations between antenatal depression and variables of sociodemographic and gestational characteristics among pregnant Omani women.

Variables	8 – 12 weeks of pregnancy N = 302						24 – 28 weeks of pregnancy N = 272					
	Non- depressive (EPDS ≤12) (n=212) (70.2%)		Depressive (EPDS) ≥ 13 (n=90) (29.8%)		χ^2^	*p*	Non- depressive (EPDS ≤12) n=189 (69.5%)		Depressive (EPDS) ≥ 13 n=83 (30.5%)		χ^2^	*p*
	N	%	N	%			N	%	N	%		
Age (Y)					0.606	0.891	​				1.568	0.664
< 20	6	2.8	3	3.3	​	​	5	2.6	3	3.6	​	​
20 – 30	95	44.8	40	44.4	​	​	83	43.9	38	45.8	​	​
30 – 40	97	45.8	43	47.8	​	​	87	43.9	39	47	​	​
> 40	14	6.6	4	4.4	​	​	14	7.4	3	3.6	​	​
Educational level					3.59	0.163	​				7.08	*0.028*
Basic Education	24	11.3	4	4.4	​	​	22	11.6	3	3.6	​	​
Secondary Education	92	43.4	41	45.6	​	​	87	46	33	39.8	​	​
Degree or higher	96	45.3	45	50	​	​	80	42.3	47	56.6	​	​
Occupational Status					1.970	0.166	​				0.337	0.567
Employed	69	32.5	22	24.4	​	​	59	31.2	23	27.7	​	​
Unemployed	143	67.5	68	75.6	​	​	130	68.8	60	72.3	​	​
Annual household Income					3.224	0.195	​				2.00	0.367
Less than 500 OR	89	42	45	50	​	​	86	45.5	35	42.2	​	​
500 OR – 1000 OR	86	40.6	36	40	​	​	71	37.6	38	45.8	​	​
Above 1000	37	17.5	9	10	​	​	32	16.9	10	12	​	​
Housing tenure					7.723	0.0213	​				4.105	0.128
Rent house	54	25.5	24	26.7	​	​	45	23.8	25	30.1	​	​
Homeowner	92	43.4	25	27.8	​	​	79	41.8	24	28.9	​	​
Live with the husband or wife family	66	31.1	41	45.6	​	​	65	34.4	34	41	​	​
Duration of Marriage					0.926	0.819	​				7.646	0.054
Less than 2 years	41	19.3	16	17.8	​	​	38	20.1	14	16.9	​	​
Less than 5 years	42	19.8	19	21.1	​	​	37	19.6	22	26.5	​	​
Less than 10 years	73	34.4	35	38.9	​	​	53	28	12	14.5	​	​
Older than 10 years	56	26.4	20	22.2	​	​	53	28	12	14.5	​	​
Planned pregnancy					1.931	0.165	​				3.059	0.080
Yes	147	69.3	55	61.1	​	​	130	68.8	48	57.8	​	​
No	65	30.7	35	38.9	​	​	59	31.2	35	42.2	​	​
Physical Activity					1.598	0.660	​				4.793	0.188
No	108	50.9	50	55.6	​	​	97	51.3	46	55.4	​	​
30 minutes for at least 2/week	64	30.2	22	24.4	​	​	56	29.6	20	24.1	​	​
30 minutes for at least 3/week	18	8.5	10	11.1	​	​	13	6.9	20	24.1	​	​
30 minutes for at least 5/week	22	10.4	8	8.9	​	​	23	6.9	11	13.3	​	​
History of Depression					21.957	0.000	​				13.826	0.000
Yes	11	5	21	24	​	​	12	6.3	18	21.7	​	​
No	201	95	69	76	​	​	177	93.7	65	78.3	​	​
Family history of depression					3.636	0.05	​				0.606	0.436
Yes	14	6.6	12	13.3	​	​	15	7.9	9	10.8	​	​
No	198	93.4	78	86.7	​	​	174	92.1	74	89.2	​	​
Parity					0.693	0.707	​				1.164	0.559
1	49	23.1	20	22.2	​	​	45	23.8	18	21.7	​	​
2	53	25	19	21.1	​	​	41	21.7	23	27.7	​	​
3	110	51.9	51	56.7	​	​	103	54.5	42	50.6	​	​
BMI at registration					1.812	0.612	​				3.112	0.375
Underweight	14	6.6	9	10	​	​	15	7.9	6	7.2	​	​
Normal	55	25.9	20	22.2	​	​	48	25.4	19	22.9	​	​
Overweight	58	27.4	28	31.1	​	​	60	31.7	20	24.1	​	​
Obesity	85	40.1	33	36.7	​	​	66	34.9	38	45.8	​	​
Obstetric complication Previous Pregnancy					4.179	0.243	​				2.168	0.538
Preeclampsia (PE)	8	3.8	0	0	​	​	7	3.7	1	1.2	​	​
Miscarriage	50	23.6	26	28.9	​	​	46	24.3	18	21.7	​	​
Preterm	11	5.2	4	4.4	​	​	7	3.7	5	6	​	​
Mode of delivery in previous pregnancies					1.723	0.423	​				1.220	0.543
Normal Delivery (SVD)	110	51.9	54	60	​	​	97	51.3	48	57.8	​	​
Caesarean section (CS)	63	29.7	23	25.6	​	​	56	29.6	23	27.7	​	​
No previous pregnancies	39	18.4	13	14.4	​	​	36	19	12	14.5	​	​

Abbreviations: BMI, body mass index; SD, standard deviation.

At 24–28 weeks, educational level emerged as a significant factor (χ^2^ (2) = 7.08, p = 0.028), with a small effect size (Cramér’s V = 0.16), indicating a slight inverse relationship between education and depression symptoms. History of depression remained significantly associated with antenatal depression (χ^2^ (1) = 13.83, p < 0.001) with a medium-to-large effect size (Cramér’s V = 0.23).

Other variables including maternal age, occupation, family income, marital duration, family history of depression, parity, past obstetric complications, mode of previous delivery, and BMI at registration—were not significantly associated with prenatal depression.

Multivariate logistic regression analysis (Table 2) identified three key predictors of antenatal depression. History of depression was the strongest predictor, increasing the odds of depression 5.4 times at 8–12 weeks [AOR = 5.42; 95% CI: 2.09–14.08] and 7.2 times at 24–28 weeks [AOR = 7.19; 95% CI: 2.50–20.70]. Surprisingly, university education was associated with a higher risk of depression, with 6.5 times higher odds at 8–12 weeks [AOR = 6.55; 95% CI: 1.70–25.13] and 8.1 times at 24–28 weeks [AOR = 8.13; 95% CI: 1.84–35.82]. In contrast, unplanned pregnancy appeared protective, reducing the odds of depression by half at both time points [AOR = 0.50 and 0.40, respectively].

**Table 2.** Logistic regression analysis of risk factors for depression symptoms among pregnant Omani women at week 8-12 and week 24 -28 of pregnancy in the study Odds Ratio (95% CI).

​	At weeks 8-12 of pregnancy	*p*	At weeks 24-28 of pregnancy	*P*
	OR (95% C.I.)		OR (95% C.I.)	
Degree or higher education level	6.55(1.70-25.13)	0.01	8.13(1.84-35.82)	0.01
Is the pregnancy planned? (No)	0.50(0.26-0.96)	0.04	0.40(0.20-0.81)	0.01
History of depression (yes)	5.42(2.09-14.08)	0.00	7.19(2.50-20.70)	0.00

Variable(s) entered in Step 1: Age categories, educational level, occupational status, annual household income, housing tenure, duration of marriage, and whether the pregnancy was planned. History of depression, family history of depression, physical activity, parity category, previous pregnancy complications, mode of delivery in previous pregnancies, BMI in registration category.

Abbreviations: OR, odds ratio; CI, confidence interval.

### 3.2. Prevalence of anxiety symptoms

Table 3 presents the prevalence of anxiety symptoms and their associations with sociodemographic and gestational factors among pregnant Omani women. Using a cut-off score of ≥6 on the 3-item anxiety subscale of the Edinburgh Postnatal Depression Scale (EPDS-3A), 24.8% of participants exhibited anxiety symptoms at 8–12 weeks, increasing slightly to 26.1% at 24–28 weeks of pregnancy.

**Table 3.** Associations between antenatal anxiety and sociodemographic gestational characteristics variables among pregnant Omani women.

Variables	8 – 12 weeks of pregnancy N = 302						24 – 28 weeks of pregnancy N = 272					
	Non-anxiety (EPDS-3A ≤ 5) n=227 (75.2%)		Anxiety (EPDS-3A ≥ 6) n=75 (24.8%)		χ^2^	*p*	Non-anxiety (EPDS-3A ≤ 5) n=75 (73.9%)		Anxiety (EPDS-3A 6) n=71 (26.1%)		χ^2^	*P*
	N	%	n	%			n	%	n	%		
Age (Y)					1.062	0.781	​				2.409	0.492
< 20	8	3.5	1	1.3	​	​	5	2.5	3	4.2	​	​
20 – 30	100	44.1	35	46.7	​	​	89	44.3	32	45.1	​	​
30 – 40	105	46.3	35	46.7	​	​	92	45.8	34	47.9	​	​
> 40	14	6.2	4	5.3	​	​	15	7.5	2	2.8	​	​
Educational level					1.831	0.400	​				5.062	0.080
Basic Education	20	8.8	8	10.7	​	​	21	10.4	4	5.6	​	​
Secondary Education	105	46.3	28	37.3	​	​	94	46.8	26	36.6	​	​
Degree or higher	102	44.9	39	52	​	​	86	42.8	41	57.7	​	​
Occupational Status					0.030	0.862	​				0.523	0.469
Employed	69	30.4	22	29.3	​	​	63	31.3	19	26.8	​	​
Unemployed	158	69.6	53	70.7	​	​	138	68.7	52	73.2	​	​
Retired	​	​	​	​	​	​	​	​	​	​	​	​
Annual household income					1.683	0.431	​				2.937	0.230
Less than 500 OR	98	43.2	36	48	​	​	90	44.8	31	43.7	​	​
500 OR – 1000 OR	91	40.1	31	41.3	​	​	76	37.8	33	46.5	​	​
Above 1000	38	16.7	8	10.7	​	​	35	17.4	7	9.9	​	​
Housing tenure					3.181	0.204	​				4.286	0.117
Rent house	58	25.6	20	26.7	​	​	47	23.4	23	32.4	​	​
Homeowner	94	41.4	23	30.7	​	​	83	41.3	20	28.2	​	​
Live with the husband or wife family	75	33	32	42.7	​	​	71	35.3	28	39.4	​	​
Duration of Marriage					4.111	0.253	​				3.855	0.278
Less than 2 years	45	19.8	12	16	​	​	36	17.9	16	22.5	​	​
Less than 5 years	49	21.6	12	16	​	​	42	20.9	17	23.9	​	​
Less than 10 years	74	32.6	34	45.3	​	​	69	34.3	27	28	​	​
older than 10 years	59	26	17	22.7	​	​	54	26.9	11	15.5	​	​
Planned pregnancy					4.112	*0.043*	​				0.511	0.475
Yes	159	70	43	57.3	​	​	134	66.7	44	62	​	​
No	68	30	32	42.7	​	​	67	33.3	27	48	​	​
Physical Activity					2.537	0.469	​				7.601	*0.05*
No	114	50.2	44	58.7	​	​	99	49.3	44	62	​	​
30 minutes for at least 2/week	69	30.4	17	22.7	​	​	63	31.3	13	18.3	​	​
30 minutes for at least 3/week	20	8.8	8	10.7	​	​	15	7.5	9	12.7	​	​
30 minutes for at least 5/week	24	10.6	6	8	​	​	24	11.9	5	7	​	​
History of Depression					15.346	*0.000*	​				7.392	*0.000*
Yes	15	6.6	17	22.7	​	​	16	8	14	19.7	​	​
No	212	93.4	58	77.3	​	​	185	92	57	80.3	​	​
Family history of depression					0.066	0.797	​				0.017	0.897
Yes	19	8.4	7	9.3	​	​	18	9	6	8.5	​	​
No	208	91.6	68	90.7	​	​	183	91	65	91.5	​	​
Parity					0.715	0.700	​				0.328	0.849
1	54	23.8	15	20	​	​	45	22.4	18	25.4	​	​
2	55	24.2	17	22.7	​	​	47	23.4	17	23.9	​	​
3+	118	52	43	57.3	​	​	109	54.2	36	50.7	​	​
Obesity BMI					1.180	0.758	​				1.029	0.794
Underweight	18	7.9	5	6.7	​	​	15	7.5	6	8.5	​	​
Normal	53	23.3	22	29.3	​	​	50	24.9	17	23.9	​	​
Overweight	65	28.6	21	28	​	​	62	30.8	18	25.4	​	​
Obesity	91	40.1	27	36	​	​	74	36.8	30	42.3	​	​
Obstetric complication Previous Pregnancy	​				4.084	0.253	​				1.100	0.777
Preeclampsia (PE)	8	3.5	0	0	​	​	7	3.5	1	1.4	​	​
Miscarriage	53	23.3	23	30.7	​	​	47	23.4	17	23.9	​	​
Preterm	12	5.3	3	4	​	​	8	4	4	5.6	​	​
Mode of Delivery in previous pregnancies					1.069	0.586	​				0.365	0.833
Normal Delivery (SVD)	121	53.3	43	57.3	​	​	109	54.2	36	50.7	​	​
Caesarean section (CS)	64	28.2	22	29.3	​	​	58	28.9	21	29.6	​	​
No previous pregnancies	42	18.5	10	13.3	​	​	34	16.9	14	19.7	​	​

Abbreviations: EPDS, Edinburgh Postnatal Depression Scale.

Univariate analysis revealed several significant associations. At 8–12 weeks, pregnancy planning was modestly associated with anxiety symptoms (χ^2^ (1) = 4.11, p = 0.043), with a small effect size (Cramér’s V = 0.12), indicating that unplanned pregnancies may slightly elevate early pregnancy anxiety levels.

At 24–28 weeks, physical activity was significantly associated with anxiety symptoms (χ^2^ (3) = 7.60, p = 0.05), with a small to medium effect size (Cramér’s V = 0.17). This suggests that women who engaged in higher levels of physical activity were less likely to report anxiety symptoms during the second trimester. Additionally, a history of depression continued to show a significant association with anxiety symptoms at this stage (χ^2^ (1) = 7.39, p < 0.001), with a medium effect size (Cramér’s V = 0.23), underlining the persistent impact of prior mental health conditions.

Multivariate logistic regression (Table 4) identified four key predictors of anxiety symptoms during pregnancy. History of depression was the strongest predictor, increasing the odds of anxiety symptoms by nearly sixfold at 8–12 weeks [AOR = 5.92; 95% CI: 2.27–15.78] and by fourfold at 24–28 weeks [AOR = 4.13; 95% CI: 1.56–10.93]. Educational level also played a role, with university-educated women showing 3.4 times higher odds of anxiety symptoms at 24–28 weeks [AOR = 3.4; 95% CI: 0.91–13.17] compared to those with secondary education.

**Table 4.** Logistic regression analysis of risk factors for anxiety symptoms among pregnant Omani women at week 8-12 and week 24 -28 of pregnancy in the study Odds Ratio (95% CI).

​	At weeks 8-12 of pregnancy		At weeks 24-28 of pregnancy	
	OR (95% C.I.)	*P*	OR (95% C.I.)	*P*
Degree or higher education level	1.69(0.54-5.27)	0.36	3.46(0.91-13.17)	0.05
Whether the pregnancy was planned? (No)	0.46(0.24-0.88)	0.02	0.71(0.36-1.40)	0.32
History of depression (Yes)	5.98(2.27-15.78)	0.00	4.13(1.56-10.93)	0.00
physical activity for 30 minutes for at least 2/week	0.74(0.37-1.51)	0.41	0.44(0.20-0.96)	0.04

Variable(s) entered in Step 1: Age categories, educational level, occupational status, annual household income, housing tenure, duration of marriage, and whether the pregnancy was planned. History of depression, family history of depression, physical activity, parity category, previous pregnancy complications, mode of delivery in previous pregnancies, BMI in registration category.

Abbreviations: OR, odds ratio; CI, confidence interval.

Interestingly, unplanned pregnancies were associated with a significantly lower risk of anxiety symptoms at 8–12 weeks [AOR = 0.46; 95% CI: 0.24–0.89]. Lastly, low physical activity levels (fewer than two sessions of 30 minutes per week) were associated with lower odds of anxiety symptoms at 24–28 weeks [AOR = 0.45; 95% CI: 0.21–0.96]. This finding should be interpreted with caution because it differs from the direction commonly reported in literature.

### 3.3. Pregnancy outcomes

Table 5 shows the pregnancy and neonatal outcomes of the study participants (n = 242). In the current pregnancy, among all participants in the study, 19% had GDM, and 11.6% had preterm delivery. The mean gestation age in days at delivery was 270.8 days. Around 13% of the pregnant women had a LBW delivery (LBW <2500 g), and 7% had delivered small babies for their gestational age. The mean length of the infant (cm), head circumference (cm) and birth weight (gm) at delivery were 49.8 cm, 33.4 cm and 2984 gm, respectively. Finally, the mean range of the Apgar score at 1 and 5 minutes was 8.8 and 9.9, respectively. Table 6 shows the associations between depression and anxiety symptoms at weeks 8-12 and 24-18 of pregnancy, with gestational duration in weeks and the birthweight centile in the linear regression analyzes.

**Table 5.** Pregnancy and neonatal outcomes of the Omani pregnant women in the study.

Variables	N (242)	%
GDM	59	19.2
Normal Delivery (SVD)	171	70.5
Caesarean section (CS)	71	29.5
Full term delivery	214	88.4
Preterm delivery	28	11.6
BMI at delivery		
Underweight	23	7.6
Normal	75	24.8
Overweight	86	28.5
Obesity	118	39.1
Low birth weight		
Low weight LBW <2500 g	30	12.4
Small Gestational Age	17	7
Average Gestational Age	208	86
Large Gestational Age	17	7
Gestational weight gain category		
Adequate	105	43.4
Inadequate	120	49.6
Excessive	17	7

​	Mean	SD
Gestation age by days at delivery	270.85	12.1
Body Mass Index (BMI) at delivery	31.5	6.76
Infant length (cm)	49.85	3.177
Birth weight (gm)	2984.31	527.7
Head circumference (cm)	33.41	1.80
Apgar score at 1 minute	8.8	0.77
Apgar score at 5 mins	9.9	0.46

Abbreviations: BMI, body mass index; SD, standard deviation.

**Table 6.** Associations between depression and anxiety symptoms with GDM, preterm and PE (N = 242).

​	GDM			S Preterm			PE		
	N=59	OR (95%CI)	*p*	N=16	OR (95%CI)	N=*p*	13	OR (95%CI)	*p*
**Depression at 8 – 12 weeks of Pregnancy**									
No	45	-	​	21	-	​	10	-	​
Yes	14	1.46 (.76-2.83)	0.25	7	0.93 (.31-2.8)	0.89	3	2.4 (.52-11.09)	0.25
**Depression at 24– 28 weeks of Pregnancy**									
No	43	-	​	22	-	​	9	-	​
Yes	10	2.15 (1.02-4.52)	0.04	6	1.33 (.41-4.28)	0.62	4	1.49 (.39-5.5)	0.55
**Anxiety at 8 – 12 weeks of Pregnancy**									
No	46	-	​	24	-	​	10	-	​
Yes	13	1.21 (.61-2.39)	0.57	4	1.4 (.4 -5.2)	0.49	3	1.85 (.4-8.58)	0.42
**Anxiety at 24– 28 weeks of Pregnancy**									
No	44	-	​	22	-	​	9	-	​
Yes	9	1.93 (.88-4.19)	0.09	6	1.06 (.33-3.41)	0.11	4	1.99 (.43-9.2)	0.37

Abbreviations: OR, odds ratio; CI, confidence interval.

Regression models (Table 7) show that the appearance of antenatal depression and anxiety symptoms is correlated with a higher probability of having a lower gestational age at delivery. For example, lower gestational age was associated with depression symptoms at 24 – 28 weeks of pregnancy (P = 0.001) (β = 0.68; 95% CI 0.013 to 1.290) and anxiety symptoms at 8 – 12 weeks of pregnancy (P = 0.02) (β = 0.75; 95% CI 0.106 to 1.3) and at 24 – 28 weeks of pregnancy (P=0.002) (β = 0.98 95% CI 0.343 to 1.616).

**Table 7.** Liner regression investigated the association between depressive and anxiety symptoms with gestational duration (days) and customised birthweight centile.

​	Gestational age by (weeks)		Customised birthweight centile	
	*β* (95%CI)	*p*	*β* (95%CI)	*p*
Depression at 8 – 12 weeks of pregnancy				
No	-	​	-	​
Yes	0.475 (-0.136-1.086)	0.196	0.008 (-7.343-8.267)	0.907
Depression at 24– 28 weeks of pregnancy				
No	-	​	-	​
Yes	0.676 (0.013-1.290)	0.003	0.8 (-6.922-8.845)	0.81
Anxiety at 8 – 12 weeks of pregnancy				
No	-	​	-	​
Yes	0.751 (0.106-1.39)	0.023	0.914 (-7.8-8.706)	0.914
Anxiety at 24– 28 weeks of pregnancy				
No	-	​	-	​
Yes	0.98 (0.343-1.616)	0.003	0.042 (-5.548 -11.025)	0.516

Abbreviations: OR, odds ratio; CI, confidence interval.

## 4. Discussion

### 4.1. Prevalence of depression and anxiety symptoms

In this prospective cohort study of 302 pregnant Omani women, the prevalence of depressive symptoms was 29.8%, while anxiety symptoms were present in 24.8% of participants in early pregnancy. The prevalence of depressive symptoms in this cohort of pregnant Omani women was higher than previously reported national estimates 12%^14^ and 24.3%,^15^ and comparable to rates found in Gulf countries with similar cultural contexts.^9,28^ It also exceeded prevalent rates in high-income countries such as the United States (16.6%), Australia (16.9%), Brazil (13.5%), and Turkey (21.6%),^29–33^ but aligned with those in parts of Asia and Africa, including China (28.5%), India (35.7%), North Tanzania (33.8%), and Northwest Ethiopia (31.2%).^34–37^ In Europe, comparable prevalence rates have also been reported. A prospective cohort study from Finland found depressive symptom prevalence of 22.5%, 22.1%, and 24.1% across the three trimesters, with an overall mean prevalence of 22.9%.^38^

Anxiety symptoms were reported in 24.8% and 26.1% of participants at two assessment points, based on the EPDS-3A cut-off of 6.^39^ These rates were similar to the 25% prevalence found in a global meta-analysis^40^ and higher than estimates from Europe (15–18%),^41^ but lower than those reported during the COVID-19 pandemic 34% in Egypt,^42^ 29% in China,^43^ and 57% in Canada.^44^

Differences in measurement tools, study design, and sociodemographic factors may explain variation across studies. The elevated prevalence in this study likely reflects the timing during the COVID-19 lockdown in Oman, a period associated with increased mental health burden in pregnancy.^42,44^ Consistent with previous findings, depression and anxiety symptoms slightly increased across the two gestational time points assessed.^45^

### 4.2. Predictors of depression and anxiety symptoms

In this cohort of pregnant Omani women, key factors significantly associated with antenatal depression included type of accommodation, history of depression, unplanned pregnancy, and educational level. Multivariate analysis further identified unplanned pregnancy, physical activity, education level, and prior depression as the strongest predictors of elevated anxiety symptoms.

Living with extended family may reflect broader household and social stressors, including crowding, limited privacy, interpersonal demands, and socioeconomic pressures, which may contribute to depressive symptoms during pregnancy.^46,47^ Additional caregiving responsibilities such as caring for ill family members may also increase maternal stress and decrease marital satisfaction, a known risk factor for depression.^48^

A history of depression was a consistent predictor of both depression and anxiety symptoms in this study, in line with earlier research.^49^ However, underreporting of past mental health issues is common in Oman due to stigma. Many women conceal out symptoms of shame and instead seek spiritual support.^50^

Surprisingly, higher educational attainment was linked to increased depressive and anxiety symptoms, mirroring results from other studies.^51^ Although highly educated women may be more aware of mental health issues, they may also experience greater internal pressure, contributing to distress.^52^ Yet, the exact mechanism for this association remains unclear.

Interestingly, unplanned pregnancy was associated with lower odds of both depressive and anxiety symptoms in the present study. This finding contrasts with much of the existing literature, where unplanned pregnancy is generally associated with increased psychological distress during pregnancy.^53^ One possible explanation is that cultural and family support systems in Oman may mitigate some of the emotional challenges associated with an unplanned pregnancy.^15^ Nevertheless, this unexpected finding should be interpreted cautiously and warrants further investigation into larger studies.

### 4.3. Pregnancy outcomes

This study found a significant association between antenatal depression at 24–28 weeks and gestational diabetes mellitus (GDM). This aligns with recent meta-analyses and reviews that highlight the link between prenatal depression and increased GDM risk.^54–56^ GDM is associated with adverse maternal mental health outcomes and developmental risks in offspring, such as cognitive and learning impairments. Additionally, depression is prevalent in up to 25% of individuals with type 2 diabetes.^57^ While the causal mechanism remains clear, emerging evidence suggests a shared inflammatory pathway, where cytokines related to depression and diabetes impair pancreatic β-cell function and promote insulin resistance.^58^ These cytokines may also activate the hypothalamic-pituitary-adrenal axis, increasing stress reactivity. Furthermore, depressive symptoms may contribute to poor diet and physical inactivity, thereby raising diabetes risk.^59^

The study also showed that maternal depression and anxiety symptoms were associated with a reduction in gestational age by approximately one week. The strongest effect was observed in anxiety symptoms at 24–28 weeks, which nearly doubled the risk of earlier delivery compared to controls. However, the lack of significant associations between anxiety/depression and preeclampsia (PE) or preterm birth contrasts with prior studies.^60,61^ Likely due to the small number of PE (n=13) and preterm birth cases (n=13). Larger studies are needed to confirm these associations in the Omani population.

No significant relationship was found between maternal mental health symptoms and small-for-gestational-age (SGA) infants. Although prior evidence supports a potential link between mid-pregnancy depression and increased SGA risk.^62^ This study used population centiles rather than customized centiles, which may underdetect true SGA cases.^63^ Previous research shows that infants identified as SGA by customized methods exhibit higher morbidity compared to those classified solely by population norms.^63,64^ Given the small number of SGA cases in this study, further well-powered research is necessary to validate these findings.

### 4.4. Strengths and limitations

The strength lies in its ability to collect reports of antenatal depression and anxiety symptoms during early pregnancy, ensuring that the report was not influenced by pregnancy outcomes. However, there are a few possible limitations to this research study. First, the study relied on self-reported questionnaires for data. This could have resulted in recall bias and unreliable estimates of the prevalence of antenatal depression. Second, the study lacks definite diagnostic standards for antenatal depression. As analyses were based on complete cases without imputation, attrition bias cannot be excluded. The EPDS is primarily a screening tool and is not intended for diagnosis. Anxiety symptoms were assessed using the EPDS-3A subscale rather than a dedicated validated anxiety instrument, which may limit the precision of anxiety measurement. Third, despite our efforts to account for potential confounding variables, there is still a chance that unmeasured factors could have impacted our findings, leading to residual confounding. Lastly, pregnant women who experience multiple responsibilities and face elevated stress levels may have hesitated to participate in the research.

## 5. Conclusions

The findings indicated that maternal depression and anxiety symptoms were higher than other estimates carried out in Oman. The study results support the need for increased public health initiatives to screen and treat depression and anxiety symptoms in pregnant Omani women as a means of prevention of adverse pregnancy outcomes. Further extensive research is required to report the incidence of prenatal anxiety and depression in Oman and to better assess the causality of the association using periodic diagnostic tests to accurately detect psychiatric disorders in expectant mothers.

Given the reasonably high incidence of anxiety and depressive symptoms among pregnant Omanis in the present study, the Oman Ministry of Health should plan to apply routine screening for prenatal depression and anxiety symptoms as a form of routine maternal care services. The early diagnosis of women with depression and anxiety symptoms would allow healthcare professionals to offer support to these women and potentially decrease the rate and impact of mental health disorders and their related complications in Oman.

## Supplemental material

Supplemental material - Depressive and anxiety symptoms, their predictors, and pregnancy outcomes among Omani pregnant women: A prospective cohort study Supplemental material for Depressive and anxiety symptoms, their predictors, and pregnancy outcomes among Omani pregnant women: A prospective cohort study by Mohammed Al Sinani, Samir Al-Adawi, and Mohammed Al Meqbaali in Women’s Health.

## Data Availability

All the data are available from the corresponding author upon reasonable request.*
