# Supplementary material for: Depressive and anxiety symptoms, their predictors, and pregnancy outcomes among Omani pregnant women: A prospective cohort study
Source: Womens Health (Lond). 2026 Aug 10;22:17455057261476574. doi: 10.1177/17455057261476574 (PMC13458133; doi:10.1177/17455057261476574)
Supplement: Supplementary file 1 [file sj-pdf-1-whe-10.1177_17455057261476574.pdf]

Appendix 1 Edinburgh Postnatal Depression Scale (EPDS)

Depression and Anxiety in The Pregnant Omani Population in Relation to Their Fatty Acid Intake and Levels

EDINBURGH POSTNATAL DEPRESSION SCALE (EPDS)

We would like to know how you are feeling. Please UNDERLINE the answer which comes closest to how you have felt IN THE PAST 7 DAYS, not just how you feel today.

Here is an example, already completed.

I have felt happy:

Yes, all the time

Yes, most of the time

No, not very often

No, not at all

This would mean: "I have felt happy most of the time" during the past week. Please complete the other questions in the same way.

In the past 7 days:

1. I have been able to laugh and see the funny side of things<br>➢ As much as I always could<br>➢ Not quite so much now<br>➢ Definitely not so much now<br>➢ Not at all	6. Things have been getting on top of me<br>➢ Yes, most of the time I haven't been able to cope at all<br>➢ Yes, sometimes I haven't been coping as well as usual<br>➢ No, most of the time I have coped quite well<br>➢ No, have been coping as well as ever
2.I have looked forward with enjoyment to things<br>➢ As much as I ever did<br>➢ Rather less than<br>➢ I used to Definitely less than I used to<br>➢ Hardly at all	7. I have been so unhappy that I have had difficulty sleeping<br>➢ Yes, most of the time<br>➢ Yes, sometimes<br>➢ Not very often<br>➢ No, not at all
3.I have looked forward with enjoyment to things<br>➢ As much as I ever did<br>➢ Rather less than I used to<br>➢ Definitely less than I used to<br>➢ Hardly at all	8. I have felt sad or miserable.<br>➢ Yes, most of the time<br>➢ Yes, quite often<br>➢ Not very often<br>No, not at all
4.I have blamed myself unnecessarily when things went wrong<br>➢ Yes, most of the time<br>➢ Yes, some of the time<br>➢ Not very often<br>➢ No, never	9. I have been so unhappy that I have been crying<br>➢ Yes, most of the time<br>➢ Yes, quite often<br>➢ Only occasionally<br>➢ No, never

5. I have been anxious or worried for no good reason<br>➢ No, not at all<br>➢ Hardly ever<br>➢ Yes, sometimes<br>➢ Yes, very often	10 . The thought of harming myself has occurred to me<br>➢ Yes, quite often<br>➢ Sometimes<br>➢ Hardly ever<br>➢ Never

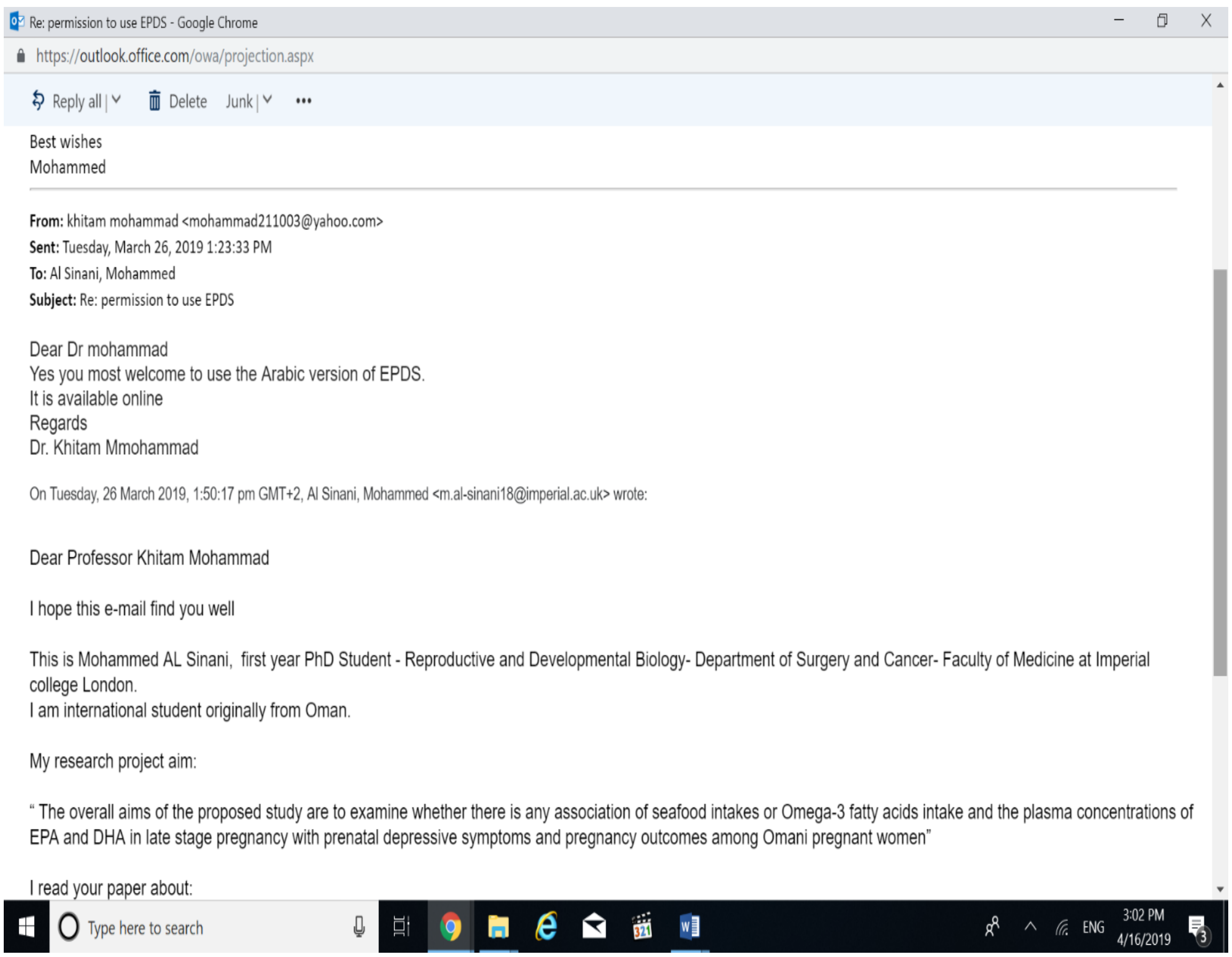

Re: permission to use EPDS - Google Chrome

https://outlook.office.com/owa/projection.aspx

Reply all | Delete Junk |

Best wishes
Mohammed

From: khitam mohammad <mohammad211003@yahoo.com>
Sent: Tuesday, March 26, 2019 1:23:33 PM
To: Al Sinani, Mohammed
Subject: Re: permission to use EPDS

Dear Dr mohammad
Yes you most welcome to use the Arabic version of EPDS.
It is available online
Regards
Dr. Khitam Mmohammad

On Tuesday, 26 March 2019, 1:50:17 pm GMT+2, Al Sinani, Mohammed <m.al-sinani18@imperial.ac.uk> wrote:

Dear Professor Khitam Mohammad

I hope this e-mail find you well

This is Mohammed AL Sinani, first year PhD Student - Reproductive and Developmental Biology- Department of Surgery and Cancer- Faculty of Medicine at Imperial college London.
I am international student originally from Oman.

My research project aim:

" The overall aims of the proposed study are to examine whether there is any association of seafood intakes or Omega-3 fatty acids intake and the plasma concentrations of EPA and DHA in late stage pregnancy with prenatal depressive symptoms and pregnancy outcomes among Omani pregnant women"

I read your paper about:

Type here to search

ENG 3:02 PM 4/16/2019

Appendix 2 Ethical Approval

Sultanate of Oman
Ministry of Health
Directorate General of Planning and Studies

سلطنة عمان
وزارة الصحة
المديرية العامة للتخطيط والدراسات

Ref. MoH/DGPS/CSR/PROPOSAL_APPROVED/22/2019 — الرقم :

Date 1.07.2019 — التاريخ :

الموافق :

Mohammed Al-Sinani
Principal Investigator

Study Title : Depressive Symptoms in Pregnant Omani Women with Low Omega-3 Fatty Acid Intake

Proposal ID: MoH/CSR/19/9668

After compliments,

We are pleased to inform you that your research proposal **'Depressive Symptoms in Pregnant Omani Women with Low Omega-3 Fatty Acid Intake'** has been approved by the Research and Ethical Review & Approval Committee, Ministry of Health.

On completion of the study, you are required to provide a copy of the final report within 2 months to the Centre of Studies and Research.

Regards,

Dr. Ahmed Mohamed Al-Qasmi
Director General of Planning and Studies
Chairman, Research and Ethical Review & Approval Committee
Ministry of Health, Sultanate of Oman.

Cc
Day file

P.O. Box : 393, Postal Code: 100, Muscat
Tel.: 22357254, Fax : 22357260
E-mail: dg.plan16@gmail.com
ص.ب : ٣٩٣ ،الرمز البريدي : ١٠٠
مسقط ، هاتف: ٢٢٣٥٧٢٥٤، فاكس : ٢٢٣٥٧٢٦٠

Appendix 3 Sociodemographic and medical characteristics of the participants

Depression and Anxiety in The Pregnant Omani Population in Relation to Their Fatty Acid Intake and Levels

General health and lifestyle questioner

Sociodemographic and medical characteristics of the participants

1. Age:

2. Educational level

	None		Basic Education		Secondary Education		Degree or above

3. Occupational status

	Employed		Unemployed		Retired

4. Annual household income

	Less than 500 OR		500 OR – 1000 OR		1000 OR or above

5. Housing tenure

	Owner house		Rent		Other

6. Smoking

	Yes		No

7. Gestation (weeks)

8. Duration of Marriage

	Less than 5 years		Less than 10 years		More than or equal to 10 years

9. Gravidity

	Primigravida		Multigravida

10. Obstetric complication Current Pregnancy

	No		Yes, please specify ……………………….…………….

11. Obstetric complication Previous Pregnancy

	No		Yes, please specify …………………………………….

12. History of Miscarriage

	Yes		No

13. Mode of Delivery in previous pregnancies

	1st trimester (0-13 week)		2nd trimester (14-27 week)		3rd trimester (28-42 week)

14. Pre-pregnancy Body Mass Index (BMI) (kg/m2)

15. Current Body Mass Index (BMI) (kg/m2)

16. Gestational weight gain (GWG)

17. Physical activity

18. Anemia status (haemoglobin levels <11.0 gm/dL),

19. History of depression

20. Family history of depression,

21. whether the pregnancy was planned or spontaneous and marital conflict.

Pregnancy Outcome:

22. Gestation Age at delivery (weeks)

23. Normal or caesarean delivery

24. Preterm birth (preterm delivery)

25. Birth sex

26. Birth recumbent length

27. Birth weight kg

28. Head circumference

29. Apgar score

30. Fetal macrosomia
